# AstraZeneca COVID-19 Vaccine and Diabetes Mellitus: A Prospective Clinical Study Regarding Vaccine Side Effects

**DOI:** 10.7759/cureus.51583

**Published:** 2024-01-03

**Authors:** Nabila Rasheed, Javeria Khan, Anusha Yusuf, Adeeba Salahuddin Khan, Arhum Mustajab, Rabika Majeed, Atif A Hashmi

**Affiliations:** 1 Department of Medicine, Basic Health Sciences, and Neuropharmacology, Sapienza University of Rome, Rome, ITA; 2 Anatomy, Dow International Dental College, Karachi, PAK; 3 Internal Medicine, Essa General Hospital, Karachi, PAK; 4 Emergency Medicine, Ziauddin University, Karachi, PAK; 5 Internal Medicine, University Hospitals of Derby and Burton NHS Foundation Trust, Derbyshire, GBR; 6 Family Medicine, Al-Samdah Health Center Duba, Ministry of Health, Duba, SAU; 7 Pathology, Liaquat National Hospital and Medical College, Karachi, PAK

**Keywords:** astrazeneca, vaccine, covid-19, swelling, fever, burning, pain, diabetes mellitus

## Abstract

Background

Coronavirus disease 2019 (COVID-19) is a major public health problem worldwide, and vaccination is currently the most effective way to control its spread and reduce its severity. Diabetes mellitus (DM) is a prevalent chronic disease that poses a significant health risk and is a frequent comorbidity in COVID-19 patients. Therefore, this study aimed to assess the frequency of local and systemic side effects of the AstraZeneca vaccine among diabetic and non-diabetic participants.

Methodology

This multicenter study was designed as a cross-sectional prospective study and was conducted in Pakistan using a non-probability consecutive sampling method. The study duration was eight months from August 1, 2022, to March 31, 2023. A total of 700 participants who received both (first and second) doses of the AstraZeneca immunization were included in the study. An independent t-test was applied to determine the association between the means and standard deviations of age, height, weight, and duration of DM and hypertension. The chi-square test was used to evaluate the association between local and systemic side effects.

Results

Among the 700 participants, 173 (49.4%) males and 177 (50.6%) females had diabetes, whereas 183 (52.3%) males and 167 (47.7%) females did not have DM; their mean ages were 46.95 ± 12.73 years (diabetics) and 38.10 ± 14.14 years (non-diabetics). The most frequent adverse effects of the AstraZeneca vaccine after the first dose were pain at the injection site, reported by 259 (74.0%) diabetics and 226 (64.6%) non-diabetic participants; however, after the second dose, injection site swelling in 170 (48.6%) diabetic and 163 (46.6%) non-diabetic recipients was the most commonly reported local side effects.

Conclusions

This study concluded that concurrent medical conditions such as DM had substantially more local and systemic side effects than those without the disease. After receiving both doses of the AstraZeneca vaccine, the most frequently reported local side effects in both diabetic and non-diabetic participants were pain, swelling, and burning at the injection site, followed by systemic side effects such as fever.

## Introduction

In late 2019, severe acute respiratory syndrome coronavirus 2 (SARS-CoV-2) was discovered for the first time. It spread quickly over the world and had a high mortality and morbidity rate. As a result, the World Health Organization (WHO) declared SARS-CoV-2 a pandemic in March 2020 [1]. SARS-CoV-2 infections can cause a wide variety of clinical signs, ranging from mild or non-existent infections to acute, potentially fatal infections affecting several organs and the respiratory system [1].

Researchers and academics from China, Great Britain, the United States, and other industrialized countries set out to develop several vaccines to halt the spread, mortality, and disastrous financial impact of coronavirus disease 2019 (COVID-19). Considering that almost 200 vaccine candidates have been studied worldwide, the development of a reliable and highly susceptible COVID-19 vaccine remains challenging [2]. Following satisfactory clinical trials, the Food and Drug Administration (FDA) of the United States, Canada, and the United Kingdom awarded permission to Pfizer/BioNTech, Moderna, and AstraZeneca vaccines [3]. The COVID-19 vaccines granted emergency use authorization include Pfizer/BioNTech, Oxford Astra-Zeneca, Moderna, Sinovac, and Sputnik V. The main countries that have offered vaccination authorization include the United States, Russia, China, the United Kingdom, and India [4].

The Pfizer-BioNTech mRNA vaccine (BNT162b2) and the Oxford-AstraZeneca vaccine (ChAdOx1 nCoV-19) were the first vaccines approved by the global healthcare authorities, followed by Sinopharm (BBIBP-CorV) [5].

BNT162b2 was developed on mRNA coding for the spike protein produced by SARS-CoV-2 and has 95% effectiveness against symptoms of COVID-19 [6]. The AstraZeneca vaccine received authorization for use in people aged 18 and above, and it demonstrated 66% effectiveness against COVID-19 [7]. China, on the other hand, developed the Sinopharm vaccine, which comprises an inactivated variant of SARS-CoV-2 HB02 with a 79% efficiency [5]. So far, no vaccine is entirely devoid of adverse reactions. Vaccination provides some level of protection against COVID-19 infection irrespective of developing adverse events [8]. Adverse reactions of vaccines vary by vaccine type; for instance, mRNA vaccines are related to higher numbers of post-vaccination side effects compared to different vaccines [9,10].

Of note, Pfizer and Oxford-AstraZeneca are currently updating their vaccines against the recently identified variants in Germany and Finland, respectively, while Moderna has yet to receive authorization from regulators for conducting trials of improved versions of vaccines for the successful targeting of the B.1.351 variant [11].

Previous studies have reported a higher frequency of COVID-19 vaccine side effects in patients with pre-existing conditions, such as hypertension and diabetes mellitus (DM). For instance, a study reported a higher frequency of injection site pain in patients with DM (64.6%) compared with patients without DM (35.4%) [12]. Therefore, it is important to evaluate the frequency of COVID-19 vaccine side effects with pre-existing conditions, such as DM. Patients with DM are prone to develop serious symptoms following a viral infection and are almost three times more likely to succumb to COVID-19. Patients with diabetes are provided special consideration for early immunizations because they frequently have less favorable results than individuals without diabetes [13]. Several immunizations have been authorized to protect people from COVID-19 infection. The vaccines offered by Johnson & Johnson, Pfizer, Moderna, and BioNTech are believed to be effective as well as secure for diabetic patients [14].

The Oxford-AstraZeneca vaccine has resulted in extremely infrequent adverse events, including the development of blood clots. However, the favorable effects associated with this vaccine exceed the risks of developing blood clots in people with diabetes [13,15]. In addition, individuals with diabetes who receive COVID-19 immunization may have mild negative reactions that involve redness, pain, or swelling at the injection site, as well as a low-grade fever. In patients with diabetes, the major adverse responses to the COVID-19 vaccination are exceedingly infrequent [13].

There is limited evidence to support the adverse health impacts of the AstraZeneca COVID-19 vaccine because the uptake of the vaccine is frequently utilized in only a few countries. However, in Pakistan, many vaccines were enrolled, including Sinopharm, Sinovac and CanSinoBIO, AstraZeneca, Moderna and Pfizer, Sputnik, and PakVac. The rate of vaccine-related adverse effects in Pakistan was 0.27-0.79 per 1,000 vaccine recipients, which was significantly low compared to Western countries [16]. The major side effects included fever, injection site pain, and headache/body aches, as reported in Pakistan [12,16-18]. AstraZeneca vaccine uptake was relatively infrequent in Pakistan compared to more widely administered Sinopharm/Sinovac and Pfizer vaccines. Injection site pain/swelling and fever were the commonly reported side effects after the AstraZeneca vaccine in Pakistan [19]. However, in Pakistan, there is insufficient data on the adverse reactions related to COVID-19 vaccines in patients with comorbidities such as DM. Therefore, this study aimed to determine the frequency of local and systemic side effects of the AstraZeneca vaccine in participants with and without diabetes among the Pakistani population. The study focused on the vaccine side effects that develop within six weeks of vaccine administration.

## Materials and methods

This multicenter study was designed as a cross-sectional investigation and was conducted in Pakistan using a non-probability consecutive sampling method. The study duration was eight months from August 1, 2022, to March 31, 2023. Ethical approval for the study was obtained from Essa General Hospital (approval number: Essa/11/2022). Informed written consent was obtained from all participants after explaining the study objectives. All patient-identifying information was removed to maintain confidentiality. A total of 700 participants who met the inclusion criteria and had received both (first and second) doses of the AstraZeneca immunization were included in the study. Participants who did not receive both doses of the AstraZeneca vaccine, those who had received other vaccinations as an alternative to the AstraZeneca vaccine, and those who had never received the COVID-19 vaccination were excluded from the study. Participants with previous COVID-19 were also included in the study. Severely immunocompromised patients were excluded from the study. Similarly, patients on steroids for autoimmune conditions and those on chemotherapy for malignancies were excluded. Moreover, cases with missing clinical details or vaccine side effects were excluded from the analysis.

The information from the participants was gathered through a questionnaire. The study provided details on the demographics of the vaccinated individuals, including gender, age, and any pre-existing conditions such as hypertension or DM. The questionnaire included all possible local and systemic side effects of the vaccine, including pain/swelling/redness/burning at the injection site, lymph node enlargement, fever, headache, nausea, body rash, flu-like illness, anxiety, myalgia, fatigue, joint aches, chills, cough, sore throat, dyspnea, diarrhea, and chest pain. The study also described the administration of the AstraZeneca vaccine at both doses and any prior COVID-19 infection. This information was collected at the time of vaccination. In addition, the study documented any local or systemic side effects that occurred following the first and second doses of the vaccine. The study also included recording the duration of time for which the patients had been suffering from DM. The survey included a question about the participants’ overall contentment with the AstraZeneca vaccine, and their responses were recorded. All information was collected at one and six weeks after vaccination. The reason for data collection at these two points was that some side effects are relatively immediate (within one week), such as injection site pain, body rash, and fever, while others take some time to appear, such as lymphadenopathy; therefore, an interval of six weeks was added to cover these side effects.

Data were entered and analyzed using SPSS Statistics for Windows, Version 26.0 (IBM Corp., Armonk, NY, USA). Mean and standard deviations were used to express age, height, weight, hypertension, and DM duration. Demographic features, including gender, frequency, and local and general side effects, were also documented. An independent t-test was applied to determine the relationship between the means and standard deviations of age, height, weight, and duration of diabetes and hypertension. The chi-square test was used to evaluate the association between local and systemic side effects for diabetic and non-diabetic participants and assess their overall satisfaction levels. A p-value <0.05 was considered statistically significant.

## Results

A total of 700 participants were included in the study. Table 1 shows the demographic profile of the study participants with respect to the presence or absence of DM. Diabetic patients had a significantly higher age (46.95 ± 12.73 years) and weight (75.30 ± 18.94 kg) than non-diabetics (p < 0.05). Similarly, a higher proportion of diabetic participants had hypertension and a history of COVID-19 infection (p < 0.05) (Table 1).

**Table 1:** Demographic details of immunized participants (n = 700). SD: standard deviation; *: P-value significant at <0.05.

Variables		Mean ± SD/n (%)		P-value
		Diabetes mellitus		
		Yes	No	
Age (years)		46.95 ± 12.73	38.10 ± 14.14	<0.001*
Weight (kg)		75.30 ± 18.94	61.57 ± 12.74	<0.001*
Height (feet)		5.34 ± 0.71	5.16 ± 0.658	0.001*
Gender	Male	173 (49.4%)	183 (52.3%)	0.450
	Female	177 (50.6%)	167 (47.7%)	
Hypertension	Yes	298 (85.1%)	27 (7.7%)	<0.001*
	No	52 (14.9%)	323 (92.3%)	
Previous COVID-19 infection	Yes	18 (5.1%)	74 (21.1%)	<0.001*
	No	332 (94.9%)	276 (78.9%)	

The most frequent adverse effects of the AstraZeneca vaccine after the first dose were injection site pain reported by 259 (74.0%) diabetic and 226 (64.6%) non-diabetic patients, with a significant difference between them (p = 0.007), whereas swelling at the injection site was reported by 161 (46.0%) diabetic and 160 (45.7%) non-diabetic participants, with an insignificant association. Furthermore, fever was experienced by 41.4% of diabetics and 33.1% of non-diabetics, with a statistically significant association (p = 0.023). Similarly, a significant association was reported between most diabetics and non-diabetics compared with the adverse events including redness at injection site (p < 0.001), headache (p = 0.001), nausea (p < 0.001), rashes (p = 0.001), flu (p < 0.001), anxiety (p < 0.001), muscle pain (p < 0.001), fatigue (p < 0.001), joint pain (p < 0.001), chills (p < 0.001), and cough (p = 0.002)(Table 2).

**Table 2:** The distribution of side effects after the first dose of the AstraZeneca vaccine among diabetes and non-diabetes participants. *: P-value significant at <0.05.

Variables		N (%)		P-value
		Diabetes mellitus		
		Yes	No	
Pain at the site of injection	Yes	259 (74.0%)	226 (64.6%)	0.007*
	No	91 (26.0%)	124 (35.4%)	
Swelling at the site of injection	Yes	161 (46.0%)	160 (45.7%)	0.940
	No	189 (54.0%)	190 (54.3 %)	
Redness at the site of injection	Yes	69 (19.7%)	22 (6.3%)	<0.001*
	No	281 (80.3%)	328 (93.7%)	
Lymphadenopathy	Yes	87 (24.9%)	89 (25.4%)	0.862
	No	263 (75.1%)	261 (74.6%)	
Fever (temperature >37.8°C)	Yes	145 (41.4%)	116 (33.1%)	0.023*
	No	205 (58.6%)	234 (66.9%)	
Headache	Yes	18 (5.1%)	44 (12.6%)	0.001*
	No	332 (94.9%)	306 (87.4%)	
Nausea	Yes	60 (17.1%)	0 (0.0%)	<0.001*
	No	290 (82.9%)	350 (100.0%)	
Rashes	Yes	36 (10.3%)	67 (19.1%)	0.001*
	No	314 (89.7%)	283 (80.9%)	
Burning at the injection site	Yes	120 (34.3%)	118 (33.7%)	0.873
	No	230 (65.7%)	232 (66.3%)	
Flu	Yes	88 (25.1%)	45 (12.9%)	<0.001*
	No	262 (74.9%)	305 (87.1%)	
Anxiety	Yes	36 (10.3%)	118 (33.7%)	<0.001*
	No	314 (89.7%)	232 (66.3%)	
Myalgia	Yes	129 (36.9%)	75 (21.4%)	<0.001*
	No	221 (63.1%)	275 (78.6%)	
Fatigue	Yes	45 (12.9%)	126 (36.0%)	<0.001*
	No	305 (87.1%)	224 (64.0%)	
Joint pain	Yes	120 (34.3%)	67 (19.1%)	<0.001*
	No	230 (65.7%)	283 (80.9%)	
Chills	Yes	68 (19.4%)	126 (36.0%)	<0.001*
	No	282 (80.6%)	224 (64.0%)	
Cough	Yes	46 (13.1%)	22 (6.3%)	0.002*
	No	304 (86.9%)	328 (93.7%)	
Sore throat	Yes	59 (16.9%)	74 (21.1%)	0.148
	No	291 (83.1%)	276 (78.9%)	
Shortness of breath	Yes	78 (22.3%)	74 (21.1%)	0.714
	No	272 (77.7%)	276 (78.9%)	
Diarrhea	Yes	59 (16.9%)	67 (19.1%)	0.431
	No	291 (83.1%)	283 (80.9%)	
Chest pain	Yes	105 (30.0%)	126 (36.0%)	0.091
	No	245 (70.0%)	224 (64.0%)	

Similarly, the most frequent adverse effects of the second dose of the AstraZeneca vaccine reported in recipients were injection site pain (n = 111, 31.7%) in diabetic and (n = 73, 20.9%) non-diabetic participants, with a significant association (p = 0.001); however, no significant association was seen between diabetics and non-diabetics with respect to swelling and burning at the injection site. Similarly, a significant association regarding the frequency of side effects between diabetics and nondiabetics was observed with respect to lymphadenopathy (p < 0.001), headaches (p = 0.001), muscular pain (p = 0.004), fatigue (p < 0.001), joint pain (p < 0.001), cough (p < 0.001), sore throat (p < 0.001), shortness of breath (p = 0.001), and diarrhea (p < 0.001) (Table 3).

**Table 3:** The distribution of side effects after the second dose of the AstraZeneca vaccine among diabetes and non-diabetes participants. N/A: not applicable; *: P-value significant at <0.05.

Variables		N (%)		P-value
		Diabetes mellitus		
		Yes	No	
Pain at the site of injection	Yes	111 (31.7%)	73 (20.9%)	0.001*
	No	239 (68.3%)	277 (79.1%)	
Swelling at the site of injection	Yes	170 (48.6%)	163 (46.6%)	0.596
	No	180 (51.4%)	187 (53.4%)	
Redness at the site of injection	Yes	9 (2.6%)	15 (4.3%)	0.213
	No	341 (97.4%)	335 (95.7%)	
Lymphadenopathy	Yes	68 (19.4%)	126 (36.0%)	<0.001*
	No	282 (80.6%)	224 (64.0%)	
Fever (temperature >37.8°C)	Yes	91 (26.0%)	104 (29.7%)	0.273
	No	259 (74.0%)	246 (70.3%)	
Headache	Yes	101 (28.9%)	141 (40.3%)	0.001*
	No	249 (71.1%)	209 (59.7%)	
Nausea	Yes	0 (0.0%)	0 (0.0%)	N/A
	No	350 (100.0%)	350 (100.0%)	
Rashes	Yes	120 (34.3%)	104 (29.7%)	0.195
	No	230 (65.7%)	246 (70.3%)	
Burning at the injection site	Yes	138 (39.4%)	148 (42.3%)	0.442
	No	212 (60.6%)	202 (57.7%)	
Flu	Yes	42 (12.0%)	30 (8.6%)	0.135
	No	308 (88.0%)	320 (91.4%)	
Anxiety	Yes	59 (16.9%)	74 (21.1%)	0.148
	No	291 (83.1%)	276 (78.9%)	
Myalgia	Yes	74 (21.1%)	45 (12.9%)	0.004*
	No	276 (78.9%)	305 (87.1%)	
Fatigue	Yes	78 (22.3%)	37 (10.6%)	<0.001*
	No	272 (77.7%)	313 (89.4%)	
Joint pain	Yes	78 (22.3%)	141 (40.3%)	<0.001
	No	272 (77.7%)	209 (59.7%)	
Chills	Yes	69 (19.7%)	89 (25.4%)	0.071
	No	281 (80.3%)	261 (74.6%)	
Cough	Yes	27 (7.7%)	89 (25.4%)	<0.001*
	No	323 (92.3%)	261 (74.6%)	
Sore throat	Yes	36 (10.3%)	104 (29.7%)	<0.001*
	No	314 (89.7%)	246 (70.3%)	
Shortness of breath	Yes	88 (25.1%)	52 (14.9%)	0.001*
	No	262 (74.9%)	298 (85.1%)	
Diarrhea	Yes	59 (16.9%)	104 (29.7%)	<0.001*
	No	291 (83.1%)	246 (70.3%)	
Chest pain	Yes	41 (11.7%)	37 (10.6%)	0.631
	No	309 (88.3%)	313 (89.4%)	

The overall satisfaction of recipients with the AstraZeneca vaccine showed that most diabetics (n = 276, 78.9%) and non-diabetics (n = 291, 83.1%) were satisfied and happy. Remarkably, none of the participants, diabetics or non-diabetics, expressed displeasure or dissatisfaction with the vaccine, except a few, who had given no view or opinion, as shown in Table 4.

**Table 4:** The overall satisfaction with the AstraZeneca vaccine among diabetic and non-diabetic participants. *: P-value significant at <0.05.

Variables		N (%)		P-value
		Diabetes mellitus		
		Yes	No	
Overall subject level of satisfaction with the vaccine	Very satisfied	65 (18.6%)	37 (10.6%)	0.001*
	Satisfied	276 (78.9%)	291 (83.1%)	
	No opinion	9 (2.6%)	22 (6.3%)	
	Dissatisfied	0 (0.0%)	0 (0.0%)	

## Discussion

Patients with persistent illnesses, such as DM, can substantially reduce their risk of COVID-19 by receiving the SARS-CoV-2 vaccine. In different studies, patients with DM did not have any major side effects after receiving the SARS-CoV-2 vaccine [20]. In this study, following the first and second doses of the AstraZeneca vaccine, both diabetic and non-diabetic groups experienced local and general side effects. We found that participants with DM had a relatively higher frequency of side effects, such as injection site pain and fever. DM is a chronic condition that impairs the patient’s immune system, which may be the reason for higher vaccine-related side effects.

A cross-sectional study involving 254 healthcare professionals in Ethiopia revealed that the incidence of at least one side effect following the administration of the first and second doses of the AstraZeneca vaccine was 91.3% and 67%, respectively. Additionally, 63.8% and 50.4% of the participants reported injection site pain following the first and second dosages, respectively. Similarly, headaches (48.8% vs. 33.5%), fever (38.8% vs. 20.9%), myalgia (38.8% vs. 21.7%), fatigue (26% vs. 28.7%), discomfort at the injection site (27.6% vs. 21.7%), and joint pain (27.6% vs. 20.9%) were among the other side effects reported after the first and second doses of the vaccination [21]. The present study was inconsistent with the above-reported studies and revealed that the most frequent adverse events after administering the first and second doses of AstraZeneca in diabetic patients were injection site pain (74.0% vs. 31.7%), injection site swelling (46.0% vs. 48.6%), fever (41.4% vs. 26.0%), and injection site burning (34.3% vs. 39.4%). The differences may be due to the difference in the study population as the above-mentioned study included only a limited number of healthcare professionals.

According to another cross-sectional study, approximately 89.8% of healthcare workers in the Czech Republic reported experiencing pain at the injection site, fatigue (62.2%), headaches (45.6%), myalgia (37.1%), and chills (33.9%) [22]. A second trial including Saudi Arabian citizens revealed the short-term negative consequences of receiving both doses of the COVID-19 vaccine. The most common symptoms were headaches, fever, flu-like symptoms, sore injection sites, and exhaustion [23]. The above studies were partially comparable to the present study, as injection site pain (74.0%) in diabetics and (64.6%) in non-diabetics, swelling injection site (46.0%) in diabetics and (45.7%) in non-diabetics, and fever (41.4%) in diabetics and (33.1%) in non-diabetics were the most reported frequently reported side effects following the first dose of the AstraZeneca vaccine, while injection site pain (31.7%) in diabetics and (20.9%) in non-diabetics, swelling at injection site (48.6%) in diabetics and (46.6%) in non-diabetics, and burning at injection site (39.4%) in diabetics and (42.3%) in non-diabetics were reported by the recipients following the second doses.

A study conducted by Almufty et al. [24] examined the adverse effects of COVID-19 vaccinations accessible in Iraq. In this study, injection site responses were most frequently reported by Pfizer recipients, whereas weariness and fever were the most frequently reported side effects among Oxford-AstraZeneca patients. Similarly, Andrzejczak-Grządko et al. [25] investigated the adverse effects of the COVID-19 vaccine in Poland. The injection site and tiredness were the most frequently reported side effects by Oxford-AstraZeneca and Pfizer vaccination recipients, respectively. On the contrary, the present study findings do not match with the above studies, as the most prevalent adverse reactions against AstraZeneca vaccine recipients were pain, swelling, and burning at the injection site, followed by fever after both doses in patients with and without DM. The demographic differences may be the reason behind the differences between our study and the above-mentioned study.

Similarly, Kaur et al. [26] studied the negative effects of the Oxford-AstraZeneca vaccination on Indian healthcare professionals through a prospective observational study. In the study, there were more systemic adverse effects (such as fever) than local ones (such as injection site pain), and the rates were higher after the initial dosage. The above study is incompatible with our study because, in the present study, local adverse events, such as injection site pain and swelling, were more widespread than systemic side effects, such as fever, after receiving the first dose of the AstraZeneca vaccine for both diabetics and non-diabetics. Again the above-mentioned study included only healthcare professionals, which may be the difference behind this difference in findings with our study.

Participants in a study by Alghamdi et al. [27] in Taif, Saudi Arabia, also had palpitations. After receiving the second dose of the Oxford-AstraZeneca vaccine, most participants experienced palpitations. The Oxford-AstraZeneca vaccination was associated with three occurrences (two females, one male) of myocarditis or inflammation of the heart muscle, according to a different study by Singh et al. [28]. It is unclear, nevertheless, whether the immunization directly contributed to the development of myocarditis because all three instances had additional comorbidities [28]. In contrast to the preceding research, our findings revealed that out of 700 participants, 85.1% hypertensive patients had diabetes, and 7.7% hypertensive patients had no diabetes, with a mean duration of 4.70 ± 1.72 years of hypertension with diabetes. However, after receiving both doses of the AstraZeneca vaccine, no palpitations or myocarditis were observed in the participants, and no serious cases were reported.

Our study demonstrated a higher frequency of COVID-19 side effects in diabetic patients. Another aspect of the COVID-19 vaccine and DM is that studies have reported an increased risk of developing type 1 DM after COVID-19 vaccination. These patients had pre-diabetes before vaccination. Similarly, reports of developing diabetic complications, such as diabetic ketoacidosis, have also been reported [29]. Apart from rising sugar levels and diabetic complications after receiving the COVID-19 vaccine, other endocrine complications were also seen after the COVID-19 vaccination. Some authors proposed that as the vaccine has an ease of access to the small pituitary gland, cases of vaccine-induced pituitary apoplexy and hypophysitis were seen. Therefore, a high index of suspicion is advised if patients develop asthenia, polydipsia, or severe headache after vaccination [30]. It is also shown that patients with pituitary adenomas are also at higher risk of developing pituitary apoplexy [31].

This study had a few limitations. Long-term follow-up was not performed to evaluate the late-onset side effects of the vaccine. As the vaccine may have late-onset side effects, which may not be covered by our study, long-term follow-up studies are needed in our population to uncover these side effects. Moreover, as data from only a few hospitals and vaccination centers were included from a single city, the results cannot be generalized, which further emphasized the need for large-scale multicenter studies on vaccine side effects. Similarly, the association of other comorbidities, such as rheumatoid arthritis, systemic lupus erythematosus, and chronic heart and liver diseases, was excluded from the study. Therefore, we recommend large-scale studies to evaluate the association of other comorbidities with COVID-19 side effects.

## Conclusions

This study concluded that patients with concurrent medical conditions such as diabetes had substantially more local and systemic side effects than those without the disease, as diabetic participants had a higher frequency of injection site pain (74% vs. 64.6%) and fever (41.4% vs. 33.1%) after the first vaccine dose than non-diabetics. This difference can be explained by the fact that DM is a chronic condition that impairs the body’s immune system and delays healing which may lead to a higher frequency of vaccine side effects.

After receiving both doses of the AstraZeneca vaccine, the most frequently reported local side effects in both diabetic and non-diabetic participants were pain, swelling, and burning at the injection site, followed by systemic side effects such as fever. It is important to note that even in DM, the overall vaccine side effects are minor, which should not restrict diabetic patients from receiving the COVID-19 vaccine.

Furthermore, most individuals were satisfied, with some being more content with the AstraZeneca immunization. Further research into the long-term adverse effects of COVID-19 vaccines using large sample sizes is indispensable. Prospective studies should examine late-onset (>6 months) vaccine side effects, especially involving participants with comorbidities, such as malignancies, and chronic infections, such as AIDs. Our study underscores the potential side effects of AstraZeneca COVID-19 adverse effects in diabetic individuals.
